# Identifying HER2 from serum‐derived exosomes in advanced gastric cancer as a promising biomarker for assessing tissue HER2 status and predicting the efficacy of trastuzumab‐based therapy

**DOI:** 10.1002/cam4.5269

**Published:** 2022-10-08

**Authors:** Qian Li, Minzhi Lv, Lihua Lv, Nida Cao, Aiguang Zhao, Jiayan Chen, Xi Tang, Rongkui Luo, Shan Yu, Yan Zhou, Yuehong Cui, Wei Guo, Tianshu Liu

**Affiliations:** ^1^ Department of Medical Oncology, Zhongshan Hospital Fudan University Shanghai China; ^2^ Department of Biostatistics, Zhongshan Hospital Fudan University Shanghai China; ^3^ Departments of Laboratory Medicine, Zhongshan Hospital Fudan University Shanghai China; ^4^ Oncology Department I Longhua Hospital Shanghai University of Traditional Chinese Medicine Shanghai China; ^5^ Department of Medical Oncology Huadong Hospital Shanghai China; ^6^ Department of Pathology, Zhongshan Hospital Fudan University Shanghai China; ^7^ Department of Laboratory Medicine, Xiamen Branch, Zhongshan Hospital Fudan University Xiamen China

**Keywords:** exosome, gastric cancer, HER2, liquid biopsy, trastuzumab

## Abstract

**Purpose:**

This study aimed to evaluate the clinical relevance of exosomal HER2 (Exo HER2) level in assessing the tissue HER2 status and predicting the efficacy of trastuzumab treatment.

**Methods:**

In this prospective study, patients with advanced gastric cancer (AGC) from three hospitals between August 2016 to November 2020 were enrolled. The Exo HER2 level was detected by enzyme‐linked immunosorbent assay. Receiver operating characteristic curve (ROC) was drawn referring to the HER2 tissue status to assess the diagnostic value of Exo HER2. Cox proportional hazards regression and logistic regression were used to evaluate the association between Exo HER2 and progression‐free survival (PFS), overall survival (OS), and objective response rate (ORR) in patients who received trastuzumab‐based first‐line therapy.

**Results:**

In this study, 242 patients with advanced or metastatic gastric adenocarcinoma were registered. Of these, 238 AGC patients were eligible for evaluating serum‐derived exosome HER2 diagnostic value, including 114 HER2‐positive. Finally, 64 were eligible for efficacy analysis. The area under the ROC curve was 0.746. The optimal cutoff value for diagnosing tissue HER2‐positive status was 729.95 ng/ml, with a sensitivity of 66.7% and a specificity of 74.2%. In 64 patients treated with trastuzumab, higher baseline Exo HER2 level indicated better prognosis. 844 ng/ml and 723 ng/ml were the right cutoffs for distinguishing the population with superior PFS (hazard ratio [HR] = 0.41, *P* = 0.017) and OS (HR = 0.30, *P* < 0.001), respectively.

**Conclusion:**

Serum exosomal HER2 level might serve as an effective biomarker for assessing tissue HER2 status in AGC and screening the potential patients who might benefit from anti‐HER2 therapy.

## Graphical abstract

In this prospective multi‐centers study, HER2 in serum‐derived exosome (Exo HER2) from patients with advanced or metastatic gastric adenocarcinoma (AGC) were analyzed for its diagnostic value and predictive value for trastuzumab therapy. The results showed Exo HER2 is a promissing biomarker for assessing tissue HER2 status in AGC and predicting clinical benefits of trastuzumab based therapy.

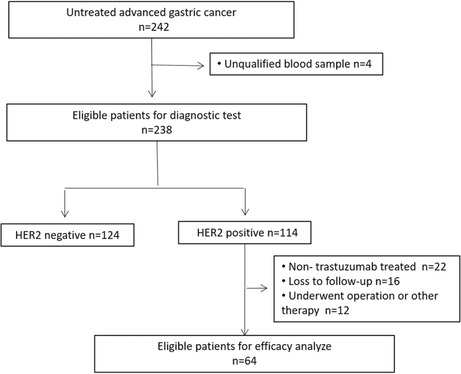

## 1 INTRODUCTION

Gastric cancer (GC) is one of the most commonly diagnosed cancers worldwide, with significant regional difference regarding its incidence.^1^, ^2^ In China, GC was the third most common cancer type and the leading cause of cancer‐related death.^3^ Human epidermal growth factor receptor‐2 (HER2) was overexpressed in 9.0%–38% of patients with advanced gastric cancer (AGC) who would benefit from trastuzumab, a humanized monoclonal antibody that targets targeted HER2.^4^, ^5^, ^6^, ^7^, ^8^ Trastuzumab for Gastric Cancer (ToGA) trial showed that trastuzumab in combination with chemotherapy resulted in a longer overall survival (OS, 13.8 months vs. 11.1 months) and progression‐free survival (PFS, 6.7 months vs. 5.5 months) compared with cisplatin plus capecitabine or cisplatin plus fluorouracil.^9^ In recent years, patients who received different regimens, including trastuzumab, also showed superior survival compared with chemotherapy alone.^10^, ^11^, ^12^, ^13^ Thus, accurate results of HER2 detection help to screen out patients with HER2 positive and improve prediction of clinical outcome of anti‐HER2 therapy. However, variations in study population ethnicity and cancer histotype, HER2 status evaluation assay utilization, and tumor heterogeneity may produce divergent outcomes in different studies.^2^ Furthermore, it is generally accepted that different detecting methods significantly affect the positive rate, especially HER2 (site and time) in gastric cancer, where the heterogeneity is higher than that in breast cancer. HER2 positivity has been reported in 22% of advanced GC but varies by tumor location and type.^14^ Therefore, HER2 status is an important consideration in deciding clinical strategies and it is urgent to establish a new method to overcome the heterogeneity of HER2 in gastric cancer.

Although immunohistochemistry (IHC) and fluorescence or silver in situ hybridization (FISH/SISH) were the standard methods used to assess HER2 overexpression in tumor tissues with significant levels of agreement,^15^, ^16^ tissue detection of HER2 still had some limitations. First, spatial intratumoral and intertumoral heterogeneities were observed in AGC.^16^, ^17^, ^18^ Intratumoral phenotypic heterogeneity was also frequently observed with a high incidence in IHC 2+ and IHC 3+ cases. Phenotypic heterogeneity was more frequently observed than genotypic heterogeneity.^14^ Previous studies showed that homogeneous overexpression was significantly associated with longer disease‐free survival compared with the heterogeneous status (20 months vs. 6 months, *p* < 0.01).^18^ Moreover, 8.7% of patients with AGC whose tumors were initially HER2 negative were identified with HER2 positivity when they underwent repeated endoscopic biopsy.^19^ HER2 positivity was lost in 29.1%–69% of patients after first‐line chemotherapy.^20^, ^21^, ^22^ Multiple biopsy or re‐biopsy was suggested, but it was difficult in most patients with AGC due to technical issues and their unwillingness. Second, the processing of tissue samples was critical. Inadequate or poor formalin fixation and delay in tissue transport led to tissue ischemia and proteolysis, resulting in inaccurate HER2 detection.^15^, ^16^ Third, HER2 detection from Cell‐free DNA (cfDNA) or circulating tumor DNA (ctDNA) was possible to be hampered in clinical practice due to the common limitation of cfDNA and ctDNA: low concentration ratio of ctDNA:cfDNA, strict analytical steps, higher input sample volume requirement, and higher costs.^23^, ^24^, ^25^ Therefore, more convenient and noninvasive detection methods are warranted to identify the HER2 status in AGC so as to screen the right patients benefiting from HER2‐targeted therapy.

Exosomes, derived from cells, contain biomolecules such as nucleic acids, proteins, and lipids; they transduce distal signals and remain stable in the blood, urine, and saliva.^26^, ^27^, ^28^, ^29^, ^30^ Exosomes provide a source of biomarkers in clinical practice due to their stability and noninvasiveness.^26^, ^27^, ^28^, ^29^, ^30^ HER2 was detected in exosomes from patients with breast cancer; also, it could be used for the early diagnosis and prognosis of patients with breast cancer in the clinic.^31^ Furthermore, HER2 was detected in exosomes in other cancers including gastric cancer^32^ and ovarian cancer.^33^ In our previous study, using a semi‐quantitative method, showed that HER2 was present in serum‐derived exosomes from patients with AGC, with levels consistent with those in tumor tissues.^32^ This multicenter study explored whether the HER2 level in serum‐derived exosomes could be used as a novel biomarker for assessing tissue HER2‐positive status in AGC and also investigated its predictive value for trastuzumab‐based therapy.

## 2 MATERIALS AND METHODS

### 2.1 Patients and samples

Patients from Zhongshan Hospital, Fudan University, Longhua Hospital, Shanghai University of Traditional Chinese Medicine, and Huadong Hospital Affiliated to Fudan University from August 2016 to November 2020 were enrolled in this study. These patients had inoperable advanced gastric adenocarcinoma and were untreated for their advanced disease. The main exclusion criteria were as follows: unavailable tumor tissue for the HER2 test using IHC or FISH; diagnosis of other malignant tumors within 5 years; or with serious infection. For efficacy analysis, some additional inclusion criteria were used as follows^1^: HER2‐positive (IHC 3+ or IHC 2+ plus FISH+) patients^2^; patients who received more than 6 weeks of trastuzumab‐based first‐line palliative chemotherapy^3^; patients with measurable lesions according to the Response Evaluation Criteria in Solid Tumors (RECIST) (version 1.1)^4^; patients with the Eastern Cooperative Oncology Group performance status of 0–2^5^; patients with left ventricular ejection fraction more than 50%; and^6^ patients with adequate bone marrow and liver and renal function. Patients with brain metastasis and receiving treatment with any other anticancer therapy were excluded from the analysis. The clinical and pathological characteristics of all enrolled patients were recorded. For the analysis of HER2 status assessment, essentially a diagnostic analysis of Exo HER2, we enrolled enough cases with HER2 positive and controls (HER2 negative) instead of consecutive patients with AGC in three hospitals.

For effective analysis, patients with HER2‐positive AGC who received trastuzumab‐based first‐line therapy with complete follow‐up information were enrolled to evaluate the relationship between the baseline exosome HER2 level and the efficacy of anti‐HER2 therapy.

### 2.2 Efficacy evaluation of trastuzumab‐based therapy

The patients enrolled for efficacy evaluation underwent computed tomography or magnetic resonance imaging according to the RECIST 1.1 criteria every 9 weeks or earlier if they had indications of treatment failure. Outcome indications included PFS, OS, and objective response rate (ORR). OS was defined as the time from the start of trastuzumab‐based treatment to the date of death from any cause. PFS was measured from the start of first‐line therapy to the date of progressive disease or death, with censoring of patients who were lost to follow‐up. ORR was defined as complete response (CR) plus partial response (PR).

### 2.3 Ethics statement

This study was approved by the research ethics committees of Zhongshan Hospital, Fudan University, Longhua Hospital, Shanghai University of Traditional Chinese Medicine, and Huadong Hospital Affiliated to Fudan University (H2019‐037). All patients signed written informed consent.

### 2.4 HER2 IHC analysis and fluorescence in situ hybridization

The classical HER2 tissue assays were performed in Shanghai Zhongshan Hospital on tissues obtained by endoscopic biopsy or on surgical specimens from the enrolled patients who underwent surgery before recurrence. IHC staining was performed with an automatic immunostainer (BenchMark XT, Ventana Medical Systems, Roche, Switzerland) using an anti‐HER2/neu antibody (4B5; pre‐dilution; Ventana Medical Systems) following the manufacturer's protocol. IHC scores of 0 and 1+ were considered as HER2 negative, whereas IHC 3+ was considered as HER2 positive. If the IHC score was 2+, FISH was performed using a PathVysion HER‐2 DNA Probe Kit (Abbott Laboratories). A FISH score >2.0 was regarded as HER2 positive. An expert pathologist confirmed the HER2 status in tumor tissues according to the guideline of the Association of Clinical Pathologists Molecular Pathology and Diagnostics Committee.^15^

### 2.5 Exosome enrichment from serum

The blood sample was prospectively collected 1–7 days before first‐line palliative therapy to isolate exosomes using ExoQuick (SBI, EXOQ5A‐1). Briefly, blood cells and debris were removed by centrifugation at 3000 rpm for 10 min and then at 13,000*g* for 15 min. Next, 250 μl of serum was incubated with 63 μl of ExoQuick exosome precipitation solution at 4°C for 30 min. The exosomes were precipitated and extracted after centrifugation at 1500*g* for 30 min.

### 2.6 Western blot analysis of exosomal protein markers

The enriched exosomes were lysed in radioimmunoprecipitation assay (RIPA) lysis buffer (Cat. P0013B, Beyotime Biotechnology) for 20 min on ice, followed by centrifugation at 14,000 rpm at 4°C for 10 min. a commercial product (Beyotime, Catalog# P0455S). Further, 10% acrylamide SDS‐PAGE gels were used for western blot analysis. We also used commercial products (Beyotime, Catalog#P0014B& P0021B) as buffers for electrophoresis and electrotransfer. Then, the samples were electrotransferred onto polyvinylidene difluoride (PVDF) membranes for 2 h. The membranes were incubated with 5% bovine serum albumin (BSA) for 1 h to exclude the binding of nonspecific antibodies and then incubated overnight with specific antibodies, including rabbit anti‐human CD9 (1:1000, Cat. 13,403 s, Cell Signaling Technology, MA, USA) CD81 (D3N2D) antibody (1:1000, Cat. #56039, CST), Calnexin (C5C9) antibody (1:1000, Cat. # 2679, CST), and TSG101 antibody (1:1000, Cat. ab125011, Abcam). Finally, the protein‐specific horseradish peroxidase‐conjugated secondary antibodies were added. The bound antibodies were detected with a BeyoECL Plus kit (Cat. P0018S, Beyotime Biotechnology) and quantified using a fluorescence imaging system (Universal Hood III, Bio‐Rad).

### 2.7 Transmission electron microscopic analysis and dynamic light scattering analysis

After being enriched by the ExoQuick (SBI, EXOQ5A‐1) kit, exosomes were resuspended in phosphate‐buffered saline (PBS) and taken for visualization by electron microscopy. Freshly isolated exosome droplets (30 μl) were fixed on the copper network for 5 min for electron microscopy. Then, the exosomes were dyed with 2% phosphotungstic acid solution for 5 min at room temperature. A dried filter paper was used to remove excess liquid, followed by heating under an incandescent lamp for 3 min. Representative pictures were photographed using a transmission electron microscope (Tecnai Spirit G2 BioTWIN, FEI). Dynamic light scattering (DLS) analysis using Nanosizer technology (Malvern Instruments) was applied for the exosome size distribution measurement.

### 2.8 Enzyme‐linked immunosorbent assay (ELISA) for detecting exosomal HER2 expression

A Human ErbB2/Her2 Quantikine ELISA Kit (R&D, DHER20) was used to assess the exosomal HER2 expression level in the serum of patients with GC following the manufacturer's protocol. Briefly, the isolated exosomes were lysed in 100 μl of cOmplete Lysis‐M ethylene diamine tetraacetic acid (EDTA)‐free medium (Roche, 04719964001), and the HER2 powder in the kit was resolved and diluted to generate the standard gradient solutions. Then, 50 μl of Assay Diluent RD1W and 50 μl of standard solution or lysate sample were added to each well and incubated for 2 h at room temperature. Next, each well was aspirated and washed with wash buffer four times. Subsequently, 200 μl of human HER2 antibody conjugate was added to each well and incubated for 2 h at room temperature on a shaker. After washing again, 200 μl of substrate solution was added and incubated for 30 min in a dark room. Finally, 50 μl of stop solution was added, and the optical density was determined within 30 min using a microplate reader set to 450 nm. The quantification of ELISA data was conducted following the manufacturer's protocol. Briefly, a standard curve was constructed by plotting the optical density (O.D.) for each standard on the *y*‐axis against the concentration on the *x*‐axis (an illustration was shown below) and a four‐parameter logistic (4‐PL) regression analysis was performed to generate the equation of O.D. on the concentration. The concentration of each sample was calculated using its O.D. and the generated equation. The upper limit was 5000 ng/ml.

### 2.9 Statistical analysis

Continuous variables were presented as mean (standard deviation) or median (interquartile range), while categorical variables were described as frequencies and percentages. The groups were compared using Student's *t* tests or Wilcoxon rank‐sum tests for continuous variables and chi‐square or Fisher's exact tests for categorical variables.

The diagnostic performance of serum exosomes for detecting HER2 was compared against the histological IHC or FISH test results as the gold standard. The sensitivity, specificity, positive predictive values, negative predictive values, and area under the receiver–operator characteristic curve (ROC) (AUC) were calculated to assess the diagnostic performance of the serum exosome level. The cutoff point of serum exosome level was determined using the Youden index. The association between survival and serum exosome level as a continuous variable was analyzed. The continuous serum exosome level was dichotomized for ease of clinical utility. The method of maximally selected rank statistics by Hothorn and Lausen was used to perform the cutoff point analysis.^34^, ^35^, ^36^ The value was chosen to assort patient outcomes according to a maximum relative risk and a minimum *p* value instead of an arbitrary selection at the median value. When the variable was dichotomized (high vs. low), the log‐rank test and Cox proportional hazards regression were used to evaluate the association between serum exosome level and survival.

All hypothesis tests were two‐sided, and a *p* value of <0.05 indicated a statistically significant difference. All data were analyzed with the R software, version 3.6.1.

## 3 RESULTS

### 3.1 Patient characteristics

**FIGURE 1:**
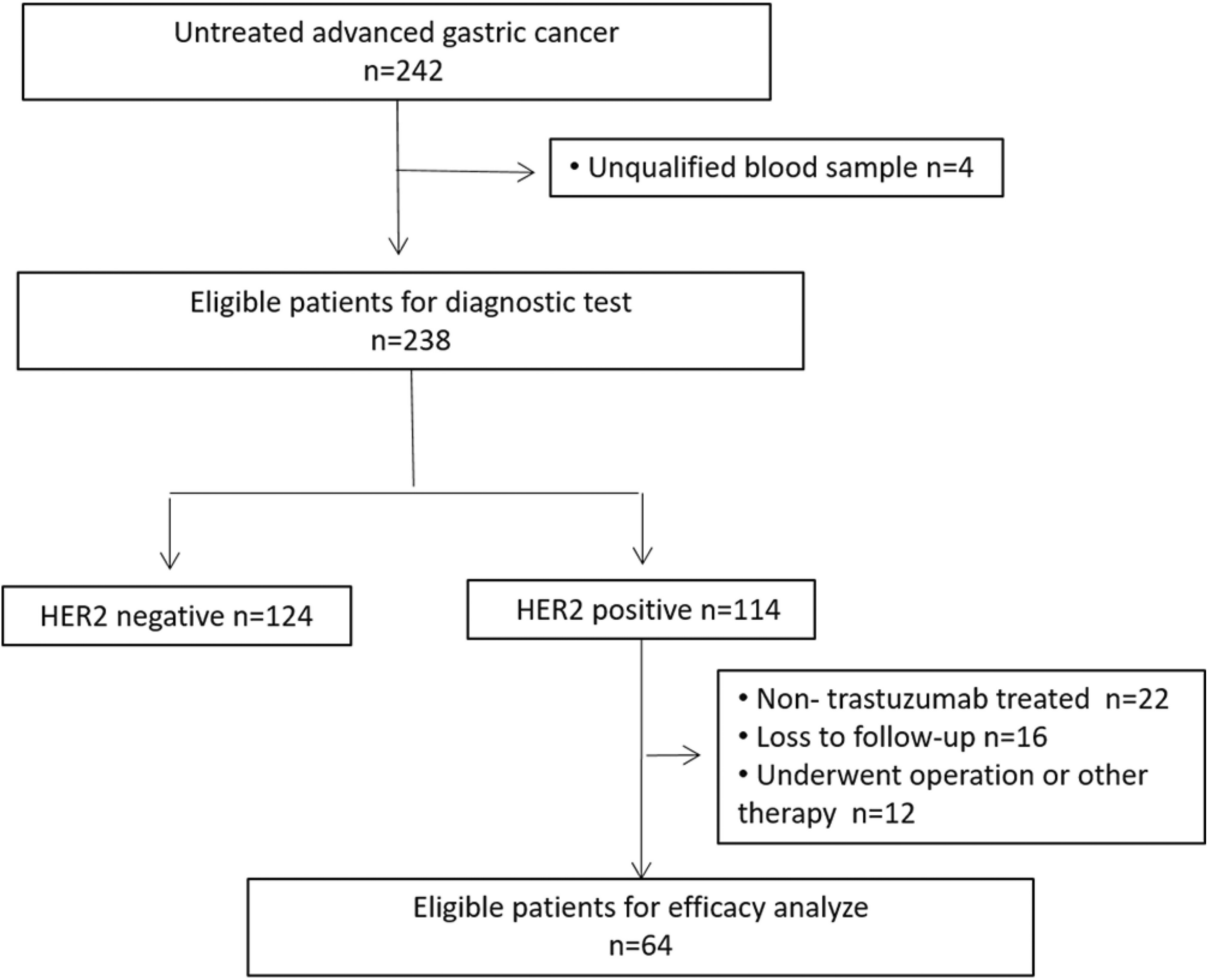
Consort diagram (trial profile).

In this prospective study, 242 patients diagnosed with advanced or metastatic gastric adenocarcinoma were registered. Of these, 238 patients were eligible for evaluating the diagnostic value of serum‐derived exosome HER2, and 64 were eligible for efficacy analysis. The Consolidated Standards of Reporting Trials diagram is summarized in Figure 1. The median age was 62 years (range 25–83). The analysis of pathological test results revealed that 114 patients were HER2 positive and 124 patients were HER2 negative. Furthermore, 41 patients had a metastatic disease involving more than 2 organs. The three most common metastatic organs were distant lymph nodes (49.6%), liver (41.2%), and peritoneum (29.0%). The interval between blood sample and pathology tissue (IBBP) was also recorded: 159 patients had an IBBP equal to or less than 180 days; IBBPs of the other 79 patients were more than 180 days. The baseline characteristics of patients are shown in Table 1.

**TABLE 1:** Baseline characteristics in AGC

	Overall (*n* = 238)	HER2 Status		*p*‐value
		Negative *n* = 124	Positive *n* = 114	
Gender, *n* (%)				
Male	154 (64.9)	70 (56.4)	84 (73.7)	0.011
Female	84 (35.1)	54 (43.6)	30 (26.3)	
Age, *n* (%)				
≤60	95 (39.9)	58 (46.9)	37 (32.5)	0.031
>60	143 (60.1)	66 (53.1)	77 (67.5)	
Primary tumor, *n* (%)				
EGJ	48 (20.2)	29 (23.4)	19 (16.7)	0.265
Gastric	190 (79.8)	95 (76.6)	95 (83.3)	
IBBP, *n* (%)				
≤180 days	159 (66.8)	93 (75.0)	66 (58.4)	0.015
>180 days	79 (33.2)	32 (25.0)	47 (41.6)	
Lauren type, *n* (%)				
Intestinal	89 (44.1)	34 (31.8)	55 (57.9)	<0.001
Non‐intestinal	113 (55.9)	73 (68.2)	40 (42.1)	
Number of metastastic organs, *n* (%)				
≤2	197(82.8)	105(84.6)	92(80.7)	0.106
>2	41(17.2)	19(15.4)	22(19.3)	
Liver metastasis, *n* (%)				
No	140 (58.8)	88 (70.9)	52 (45.5)	<0.001
Yes	98 (41.2)	36 (29.1)	62 (54.5)	
Lung metastasis, *n* (%)				
No	219 (92.0)	118 (95.2)	101 (89.1)	0.131
Yes	19 (8.0)	6 (4.8)	13 (10.9)	
Peritoneal metastasis, *n* (%)				
No	169 (71.0)	80 (64.5)	89 (78.2)	0.031
Yes	69 (29.0)	44 (35.5)	25 (21.8)	
Lymphatic metastasis, *n* (%)				
No	120 (50.4)	59 (46.4)	61 (53.5)	0.329
Yes	118 (49.6)	65 (53.6)	53 (46.5)	
Other metastasis, *n* (%)				
No	161 (67.6)	76 (61.6)	85 (74.5)	0.048
Yes	77 (32.4)	48 (38.4)	29 (25.5)	
CEA level, *n* (%)				
Normal	132 (55.5)	74 (59.7)	58 (50.9)	0.217
Elevated	106 (44.5)	50 (40.3)	56 (49.1)	

Abbreviations: CEA, carcino‐embryonic antigen; EGJ, esophagogastric junction; HER2, human epidermal growth factor receptor‐2; IBBP, interval between blood sample and pathology tissue.

### 3.2 Cutoff and diagnostic value of HER2 detection in serum‐derived exosomes for assessing tissue HER2‐positive status

**FIGURE 2:**
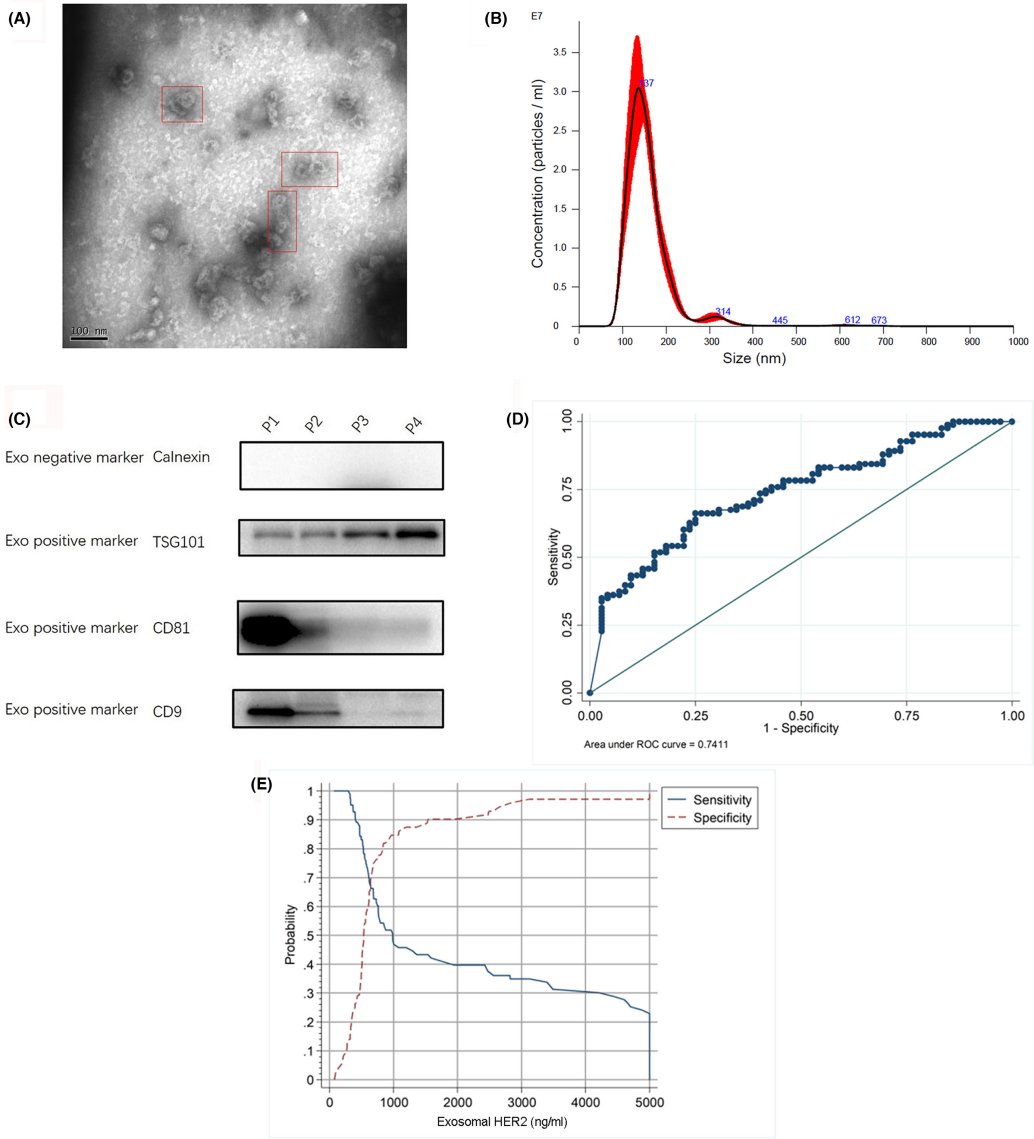
Diagnostic effect of serum exosomal HER2. (A) Isolated serum exosome image obtained using transmission electron microscopy (TEM). (B) Diameter of the particle size ranged from 50 nm to 200 nm by dynamic light scattering (DLS). (C) Expression of exosomal positive (CD9, TSG101, and CD81) and negative (calnexin) protein markers was detected using Western blot analysis. (D) Receiver operating characteristic curve of exosomal HER2. (E) Sensitivity and specificity of each cutoff point. The largest Youden index was located near the intersection of two curves.

Serum‐derived exosomes from enrolled patients were collected before palliative chemotherapy and characterized by transmission electron microscopy (TEM) and Western blot analysis. TEM revealed structurally intact exosomes with a probable diameter of 50–150 nm (Figure 2A). Besides, the DLS results showed that the particle diameter ranged from 50 nm to 200 nm, which was consistent with the diameter of exosomes (Figure 2B). Proteins specific to exosomes were evaluated by Western blot analysis using anti‐TSG101, anti‐CD81, anti‐CD9, and anti‐calnexin to confirm the presence of exosomes in the serum samples. The results showed that TSG101, CD81, and CD9 could be detected, while calnexin was negative, which confirmed the successful enrichment of exosomes (Figure 2C).

**FIGURE 3:**
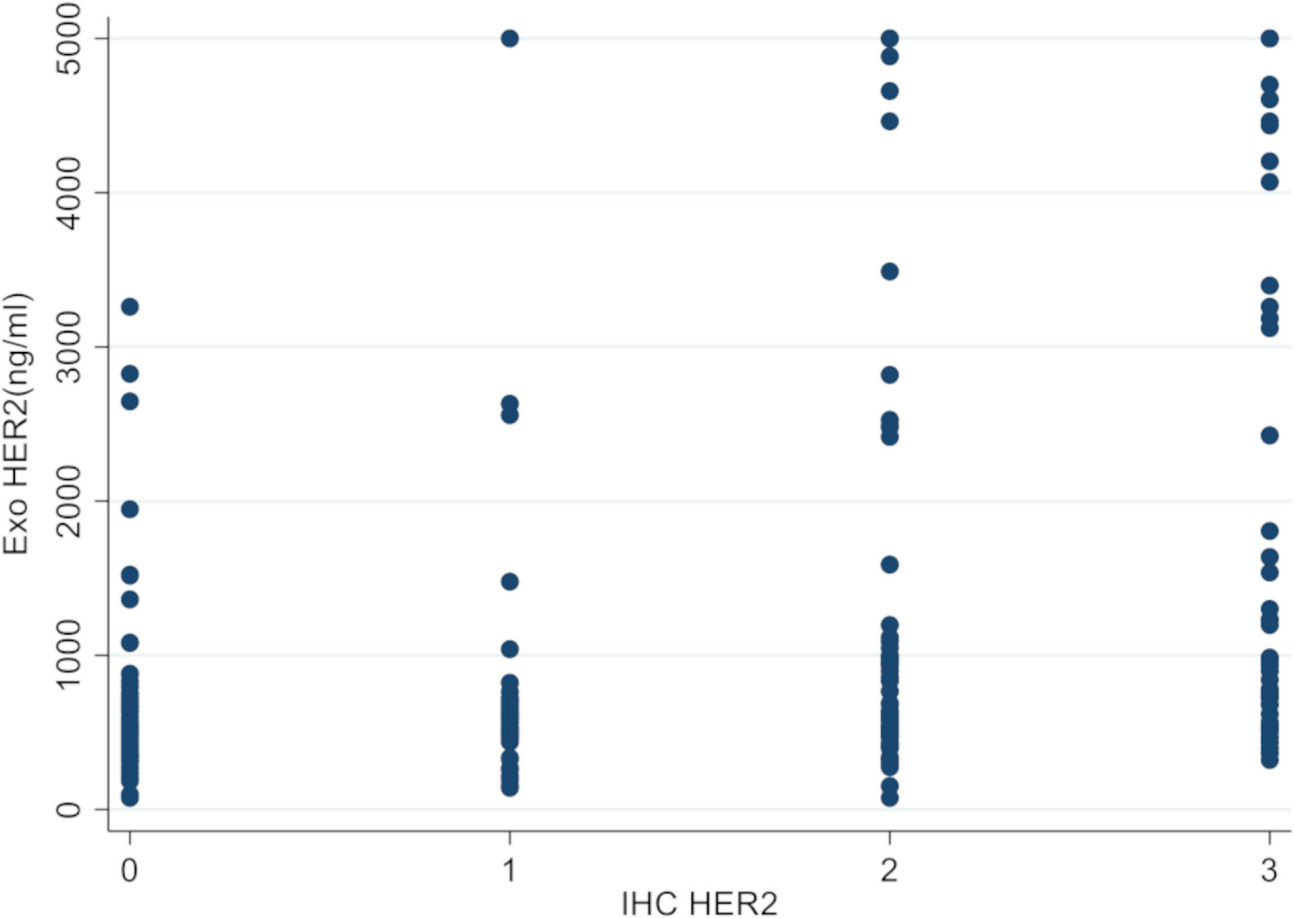
Scatter plot of the correlation between HER2 IHC status and Exo HER2 level.

**FIGURE 4:**
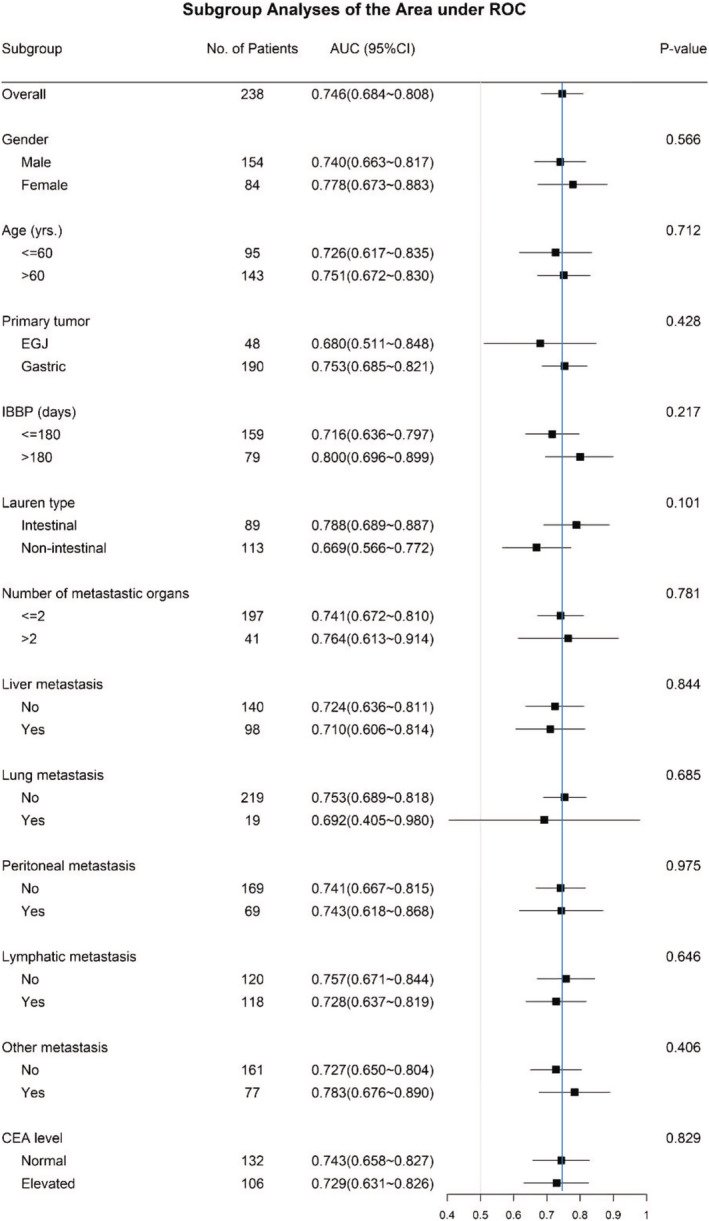
Diagnostic effect of exosomal HER2 in patients with different clinicopathological features. The blue line indicates the diagnostic effect in all enrolled patients (AUC = 0.746).

The median level of serum exosomal HER2 (Exo HER2) was 677.34 ng/ml (0–5000 ng/ml). The ROC of Exo HER2 (Figure 2d) was constructed; the area under ROC was 0.746 (95% confidence interval [CI]: 0.684–0.808), considering the tissue HER2 status as the gold standard. According to the maximum value of the Youden index, the optimum diagnostic cutoff for tissue HER2 positive was 729.95 ng/ml (Figure 2E). The sensitivity, specificity, positive predictive value, and negative predictive values of this cutoff were 66.7% (95% CI: 56.9–74.2), 74.2% (95% CI: 66.8–81.4), 70.4%, and 70.8%, respectively. The correct classification rate of the optimum diagnostic cutoff, presenting the consistency between Exo HER2 and tissue HER2 statuses, was 70.59%. After analyzing this diagnostic cutoff of Exo HER2 in patients with different IHC score, scatter plot showed a statistically significant positive correlation between HER2 IHC status and Exo HER2 level (Spearman correlation, 0.4431; *p* < 0.001) (Figure 3). In addition, the study also analyzed the diagnostic effect in patients with different clinicopathological characteristics, including gender, age, location of primary tumor, IBBP, Lauren type, number of metastatic organs, site of metastasis, and carcinoembryonic antigen (CEA) (Figure 4). No significant difference was found between subgroups, indicating that Exo HER2 had a stable effect in assessing tissue HER2 status.

### 3.3 Association between baseline Exo HER2 and outcomes of trastuzumab‐based therapy

**FIGURE 5:**
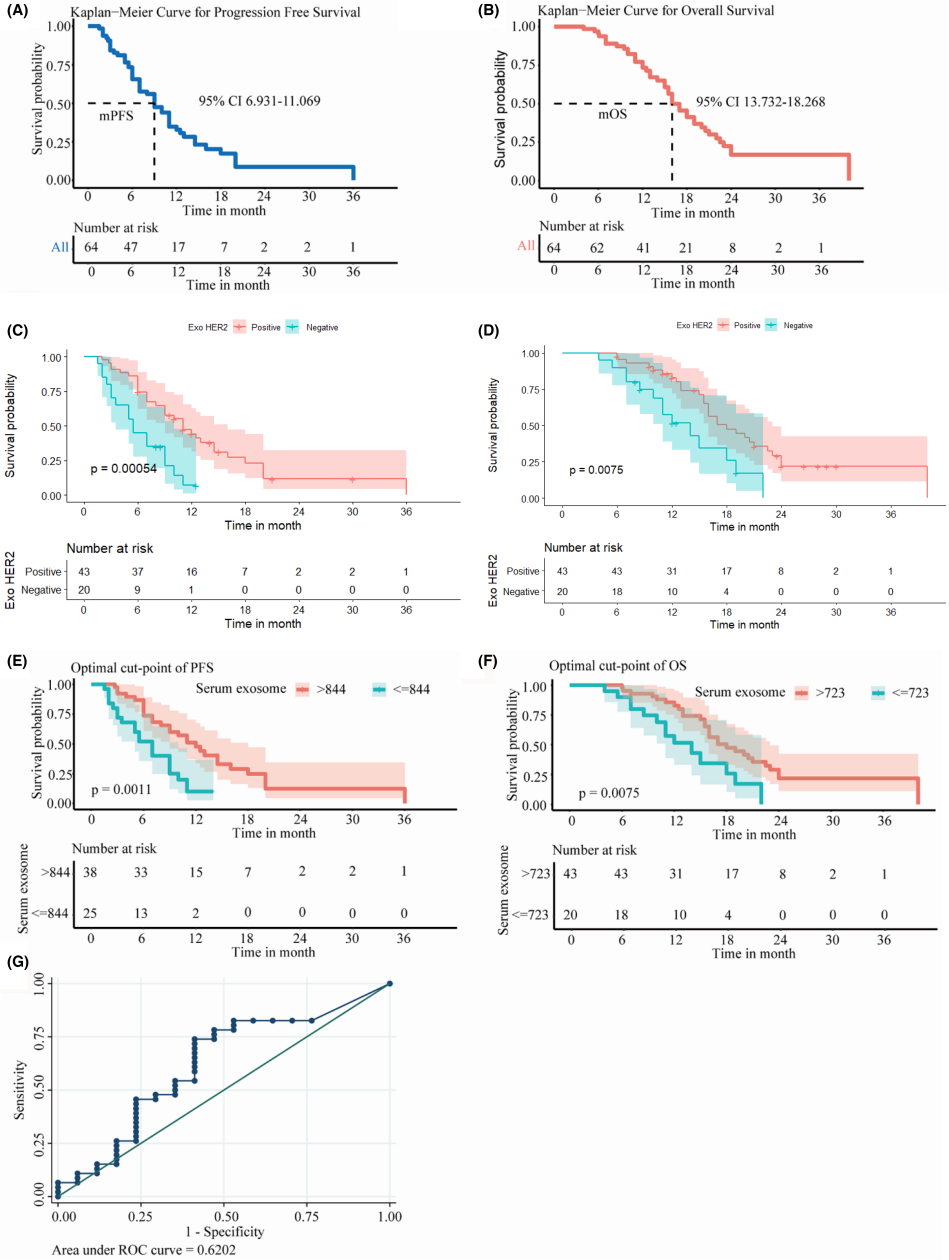
Association between the baseline Exo HER2 and outcomes of trastuzumab‐based therapy. (A) PFS curve of the 64 trastuzumab‐treated patients. (B) OS curve of the 64 trastuzumab‐treated patients. (C) PFS curve between patients with negative and positive Exo HER2 results. (D) OS curve between patients with negative and positive Exo HER2 results. (E) PFS curve between HER2‐positive patients with different Exo HER2 levels (cutoff = 844 ng/ml). (F) OS between HER2‐positive patients with different Exo HER2 levels (cutoff = 723 ng/ml). (G) Receiver operating characteristic curves of Exo HER2 for predicting ORR.

Among 238 patients, 64 with HER2‐positive AGC, who received trastuzumab‐based first‐line chemotherapy with complete follow‐up data, were eligible to analyze the relationship between Exo HER2 and outcomes. The clinicopathological and treatment‐related characteristics of patients are shown in Table 2. A total of 56 patients (87.5%) received fluoropyrimidine plus platinum or fluoropyrimidine plus taxane as the first‐line chemotherapy regimen. The other eight patients were treated with irinotecan plus cisplatin or single‐agent chemotherapy. The median number of trastuzumab treatment cycles was 12 (range 2–48). Until the last patient visit on May 15, 2021, 51 patients had disease progression; 42 patients died of tumor progression. The Kaplan–Meier curves showed that the median PFS was 9 months (95% CI 6.931–11.069) (Figure 5A) and OS was 16 months (95% CI 13.732–18.268) (Figure 5B). The confirmed response to trastuzumab in all 64 patients was as follows: 1 CR (1.5%), 17 PR (26.6%), 38 SD (59.4%), and 8 PD (12.5%). No patients discontinued treatment due to toxicity in this study; no treatment‐related death or cardiac adverse events (AEs) were reported either. The leucopenia/neutropenia (48.3%) was the most common hematological AE, while nausea was the most common nonhematological AE.

**TABLE 2:** Characteristics of 64 patients

Characteristic	*n*	%
HER2 status		
IHC 3+	39	64.06
IHC 2+	23	35.94
IHC 1+	2	3.12
Gender		
Male	49	76.56
Female	15	23.44
Age		
<60 years	42	65.63
≥60 years	22	34.37
IBBP		
≤180 days	51	79.69
>180 days	13	20.31
Tumor location		
EGJ	23	35.94
Other stomach	41	64.06
Lauren classification		
Intestinal	30	46.88
Non‐intestinal	34	53.13
WHO classification		
Well/moderately	11	17.19
Poorly/undifferentiated	53	82.81
Number of metastasis organs		
≤2	52	81.25
>2	12	18.75
Liver metastasis		
No	29	45.31
Yes	35	54.69
Peritoneal metastasis		
No	51	79.69
Yes	13	20.31
Lung metastasis		
No	57	89.06
Yes	7	10.94
LN metastasis		
No	34	53.13
Yes	30	46.88
Bone metastasis		
No	56	87.50
Yes	8	12.50
Chemotherapy regimen		
FT	16	25.00
FP	40	62.50
Others	8	12.50

Abbreviations: EGJ, Esophagogastric junction; FT: Fluorouracil plus taxane; HER2, human epidermal growth factor receptor‐2; IBBP, interval between blood sample and pathology tissue; IHC, immunohistochemistry; FP, Fluorouracil plus platinum.

First, 64 HER2 tissue‐positive patients were grouped by diagnostic cutoff (729.95 ng/ml) of Exo HER2 level. Significant difference was observed between the two groups. PFS and OS were all superior in patients with Exo HER2‐positive results than in patients with Exo HER2‐negative results (Figure 5C,D).

Second, to evaluate the predictive value of Exo HER2 in patients treated with trastuzumab‐based therapy and determine the precise cutoff to find out patients who would possibly gain benefit, the method of maximally selected rank statistics by Hothorn and Lausen was used. Since the Exo HER2 data followed non‐normal distribution, the survival prognosis was analyzed after logarithmization of the data. The results showed that the baseline Exo HER2 level had a linear relationship with OS, PFS, and ORR: the higher Exo HER2 indicated a lower risk of disease progression and death besides the higher objective response (PR + CR) rate. A baseline Exo HER2 of 844 ng/ml was obtained as the optimal cutoff using maximally selected rank statistics to distinguish the advantageous group with superior PFS (12 months [95% CI 8.848–15.152] vs. 7 months [95% CI 5.025–8.975], *p* = 0.0011) (Figure 5E). The median OS in patients whose baseline Exo HER2 was more than 723 ng/ml was 18 months (95% CI 9.033–18.967) compared with 14 months (95% CI: 13.817–18.268) in patients with lower Exo HER2 (*p* = 0.0075; Figure 5F). In addition, the ROC of Exo HER2 in distinguishing patients with ORR from non‐ORR patients was plotted (AUC = 0.620) (Figure 5G). Youden's index yielded an optimal cutoff value of 2488 ng/ml, with a sensitivity of 73.9% (95% CI: 59.7–84.4) and a specificity of 58.8% (95% CI: 36.0–78.4).

### 3.4 Univariate and multivariate analyses for Exo HER2

Univariate analysis was performed on demographic profiles, clinicopathological characteristics, and treatment features using PFS, OS, and ORR to determine whether Exo HER2 was an independent predictive or prognostic factor for trastuzumab‐based therapy. The results showed that, except for Exo HER2, Lauren type, number of metastatic sites, chemotherapy regimen, IHC of HER2, and IBBP were all unrelated to patient outcomes. Meanwhile, baseline Exo HER2 was the only independent factor associated with PFS (adjusted HR 0.41, 95% CI 0.20–0.85, *p* = 0.017) and OS (adjusted HR 0.30, 95% CI 0.15–0.58, *p* < 0.001) according to the multivariate analyses, while adjusted HR for ORR was 0.78 (95% CI 0.20–3.10) with a *p* value of 0.726 (Table 3).

**TABLE 3:** Univariate and multivariate analysis for Exo HER2.

	Crude HR	95% CI		*p*‐value	Adjusted HR^a^	95% CI		*p*‐value
Outcome: PFS								
Exo HER2‐Lower than cutoff	ref.				ref.			
Exo HER2‐Higher than cutoff	0.37	0.20	0.68	0.002	0.41	0.20	0.85	0.017
Outcome: OS								
Exo HER2‐Lower than cutoff	ref.				ref.			
Exo HER2‐Higher than cutoff	0.41	0.21	0.80	0.010	0.30	0.15	0.58	<0.001
Outcome: ORR								
Exo HER2‐Lower than cutoff	ref.				ref.			
Exo HER2‐Higher than cutoff	0.28	0.09	0.88	0.030	0.78	0.20	3.10	0.726

Abbreviations: Exo HER2, exosomal HER2; ORR, objective response rate; OS, overall survival; PFS, progression free survival.

a Model adjusted by age, gender, number of metastasis organs (>2 vs. ≤2), IHC HER2 level, chemotherapy (FP vs. FT vs. others) and IBBP (>180 days vs. ≤180 days).

## 4 DISCUSSION

Since the publication of ToGA trial, clinical treatment decisions for AGC were made depending on the results of the HER2 status. The missing HER2 data led to a worse prognosis. However, the HER2‐positive rate in China was lower than that in most other countries.^7^ In a real‐world study of 40,842 patients in China, the HER2‐positive rate was 8.8%; and HER2 heterogeneity was observed in 7.6% of patients.^37^ The HER2‐positive rate varied widely in different medical sites due to the differences in submission rates and technical competence of the pathological department. Moreover, variations in different study population ethnicity, cancer histotype, HER2 status evaluation assay utilization and tumor heterogeneity might produce divergent outcomes in sundry studies.^2^, ^38^ Anh Thu Phan et al. reported a high level of concordance between ISH and IHC analyses, an amplification rate of the HER2 gene of 15.9% and protein overexpression of the HER2 gene of 24.5%, and elevated HER2 heterogeneity in gastric cancers (68.8% phenotypic heterogeneity and 57.6% genotypic heterogeneity).^38^ HER2 positivity (overexpression [IHC3+] and/or gene amplification [FISH positive]) has been reported in 22% of advanced G/GEJ cancers but varies by tumor location and type in ToGA study, where HER2 staining (≤30% stained cells) by IHC was observed in almost 50% of cases.^14^ Intratumoral phenotypic heterogeneity was also frequently observed with a high incidence of 63.5% in IHC 2+ cases and 28.3% in IHC 3+ cases, and phenotypic heterogeneity was more frequently observed than genotypic heterogeneity (48.8% vs. 26.8%).^14^ Therefore, developing a novel, noninvasive, and simple‐operation approach as a favorable supplement is imperative for traditional tissue HER2 assessment, especially in patients with recurrent disease or unsuitable status for biopsy.

Detecting HER‐2 in exosomes in the clinical practice has several advantages. First, it is noninvasive and is able to dynamically monitor disease change, especially for patients who are unable to receive pathological tests.^30^ Second, liquid biopsy can overcome the tissue heterogeneity of HER2 expression to a certain degree and some of the limitations of solid biopsy.^39^, ^40^ Furthermore, detecting HER2 is low cost and highly repetitive, compared with second‐generation sequencing and extracting blood ctDNA. The convenience of the detection lies in extracting exosomes with test kit and testing HER2 by ELISA method. Exosomes are considered ideal drug delivery systems with a vast array of applications in various diseases, including gastric cancer. Moreover, these exosomes' anti‐tumor effect on cancer cells depends on HER2 for survival but does not affect cells that lack HER2.^41^

Baran et al. showed HER2 in cell membrane microfragments called microvesicles with stronger expression in the plasma of patients compared with healthy people.^42^ Besides, HER2 was detectable in serum‐derived exosomes using Western blot analysis, and the level was consistent with that in tumor tissues.^32^ In this study, the Exo HER2 level was determined using ELISA, a quantitative method, which made it more practicable and accurate to evaluate the diagnostic effects of Exo HER2 in AGC for assessing the HER2 status and predictive value for trastuzumab‐based therapy. According to our results, Exo HER2 might be a novel and promising liquid biopsy biomarker. A few studies were conducted to evaluate the diagnostic value of liquid biopsy biomarkers, such as circulating HER2 copies from cfDNA or ctDNA or extracellular domain in AGC^43^, ^44^, ^45^, ^46^, ^47^: AUC, a common index to measure the classifier's discriminative ability, was 0.79 with a sensitivity of 54% and specificity of 93% for ECD. The concordance rate of plasma HER2 and IHC/SISH was 63.8% (51/80), with a sensitivity of 37.5% and specificity of 90.0%. These findings indicated that the result of our study was comparable with a potential superiority of sensitivity. Additionally, only 13–39 patients with HER2‐positive AGC were reported in previous studies.^43^, ^45^, ^46^, ^48^ This deficiency inevitably weakened the accuracy of analyzing the results. Enough patients with HER2‐positive AGC (*N* = 114) were enrolled in this study to evaluate the diagnostic effect of Exo HER2 more accurately, reliably, and credibly.

Furthermore, some characteristics affecting the efficacy of liquid biopsy were analyzed. The number of metastatic organs was considered as a marker of tumor burden, while different metastatic organs were referred to as hematogenous, lymphatic, or implantation metastatic. Moreover, an IBBP defined as 180 days was used to differentiate between initial AGC and disease relapse. The results showed that the diagnostic effect of Exo HER2 in assessing tissue HER2 status was stable and equal in all subgroups.

How to screen the potential beneficiaries of trastuzumab‐based therapy is another issue in the clinical scenario of HER2‐positive AGC. The results showed that patients with a higher baseline Exo HER2 level might gain a better outcome from trastuzumab‐based therapy. Consistently, HER2 gene amplification was reported to be associated with an effective response with relatively stronger expression in HER2‐positive AGC.^49^ Our results provided a potential and promising strategy to predict the efficacy of standard anti‐HER2 therapy. Significantly, the diagnostic cutoff for tissue HER2 positive was 729.95 ng/ml, which was higher than the cutoff of 723 ng/ml for distinguishing the advantageous group with superior OS. That was serum Exo HER2 positive might indicate the patients of superior prognosis. Therefore, the clinical value of serum Exo HER2 was worth further attention. However, the number of patients with objective response to trastuzumab was rather small (*N* = 18), which might explain why Exo HER2 was not confirmed to be an independent predictive factor in multivariate analysis but was meaningful in univariate analysis.

In this study, a low‐cost and easy‐operation method was used to detect Exo HER2 in AGC and provide a potential application of Exo HER2 in clinical practice compared with ctDNA. However, this study had some limitations. First, we collected equal volumes of serum from different patients for exosome enrichment, and all exosomes were precipitated and extracted using ExoQuick protocols. Therefore, the obtained exosome concentration was relatively stable. However, we only detected serum exosomes number in a small sample with different Exo HER2 levels and found no significant difference of exosome concentration between Exo HER2 high‐level group and Exo HER2 low‐level group (Figure S1). These findings suggest that the number of serum exosomes might not affect the level of Exo HER2, which requires further validation with larger samples. Second, although this was a multi‐center study, the limitation of participating centers and non‐continuous samples might lead to some extent sample error. Third, the predictive value of Exo HER2, especially the exactly cutoff, was warranted to be confirmed in further study with a large sample size since our study only utilized 64 eligible patients during efficacy analysis. Moreover, dynamic monitoring of Exo HER2 levels during anti‐HER2 therapy could provide more information about its predictive value compared with patients' imaging evaluations.

## 5 CONCLUSION

HER2 in serum‐derived exosomes might be a highly specific surrogate biomarker for assessing the tissue HER2 status with a stable diagnostic effect in patients with AGC. Moreover, baseline serum Exo HER2 was a potential predictive marker of prognosis to screen the population who might have benefited from trastuzumab‐based therapy. Further studies in clinical settings are needed to validate the eligibility of Exo HER2 for monitoring the trastuzumab efficacy.

## AUTHOR CONTRIBUTIONS

**Qian Li:** Conceptualization (equal); data curation (equal); formal analysis (equal); investigation (equal); methodology (equal); writing – original draft (equal). **Minzhi Lv:** Conceptualization (equal); data curation (equal); formal analysis (equal); investigation (equal); methodology (equal); writing – original draft (equal). **Lihua Lv:** Data curation (supporting); formal analysis (supporting); writing – review and editing (supporting). **Nida Cao:** Data curation (supporting); formal analysis (supporting); writing – review and editing (supporting). **Aiguang Zhao:** Data curation (supporting); formal analysis (supporting); writing – review and editing (supporting). **Jiayan Chen:** Data curation (supporting); formal analysis (supporting); writing – review and editing (supporting). **Xi Tang:** Data curation (supporting); formal analysis (supporting); writing – review and editing (supporting). **Rongkui Luo:** Data curation (supporting); formal analysis (supporting); writing – review and editing (supporting). **Shan Yu:** Data curation (supporting); formal analysis (supporting); writing – review and editing (supporting). **Yan Zhou:** Data curation (supporting); formal analysis (supporting); writing – review and editing (supporting). **Yue‐Hong Cui:** Data curation (supporting); formal analysis (supporting); writing – review and editing (supporting). **Wei Guo:** Conceptualization (lead); formal analysis (lead); funding acquisition (lead); project administration (lead); writing – review and editing (lead). **Tian‐Shu Liu:** Conceptualization (lead); formal analysis (lead); funding acquisition (lead); project administration (lead); writing – review and editing (lead).

## FUNDING INFORMATION

This study was supported by the Shanghai Science and Technology Commission (17411951400) and the key medical and health projects of Xiamen (YDZX20193502000002). The funders had no role in the design of the study, in the collection, analyses, or interpretation of data, in the writing of the manuscript, or in the decision to publish the results.

## CONFLICTS OF INTEREST

The authors have no relevant financial or non‐financial interests to disclose.

## ETHICS APPROVAL

This study was performed in line with the principles of the Declaration of Helsinki. Approval was granted by the research ethics committees of Zhongshan Hospital, Fudan University, Longhua Hospital, Shanghai University of Traditional Chinese Medicine, and Huadong Hospital Affiliated to Fudan University (H2019‐037).

## CONSENT TO PARTICIPATE

Informed consent was obtained from all individual participants included in the study.

## CONSENT TO PUBLISH

Not Applicable.

## Supporting information


Figure S1

## Data Availability

The datasets generated during and/or analysed during the current study are available from the corresponding author on reasonable request.
